# *Monascus purpureus* Went rice in nephrotic hyperlipidemia

**DOI:** 10.4103/0971-4065.42334

**Published:** 2008-04

**Authors:** O. Gheith, H. Sheashaa, M. Sobh, M. Abdelsalam, Z. Shoeir

**Affiliations:** Nephrology Unit, Urology and Nephrology Center, Mansoura University, Mansoura, Egypt; 1 Neurology Department, Mansoura University, Mansoura, Egypt; 2 Internal Medicine Department, Mansoura University, Mansoura, Egypt

**Keywords:** Glomerulonephritis, *Monascus purpureus*, nephrotic syndrome, neurotoxicity, statin

## Abstract

**Background:**

Nephrotic dyslipidemia is a risk factor for the development of systemic atherosclerosis; and may aggravate glomerulosclerosis and enhance progression of glomerular disease. We aimed to assess the efficacy and safety of *Monascus purpureus* Went rice vs. fluvastatin therapy in the management of nephrotic dyslipidemia.

**Materials and Methods:**

Seventy-two patients with persistent idiopathic nephrotic syndrome (NS) with secondary dyslipidemia were included. They were randomly allocated into three age and sex-matched groups. The first group comprised 20 cases and were given *M. purpureus* Went rice in a dose of 600 mg twice/day for 1 month then once daily, the second group comprised 30 cases were given fluvastatin in a daily dose of 20 mg. The remaining 22 received no antidyslipidemic therapy and constituted a control group. All of these patients were subjected to thorough laboratory investigations including renal function tests and lipogram. Moreover, the neuromuscular status was evaluated with electromyography and nerve conduction velocity.

**Results:**

Our results showed that both fluvstatin and *M. purpureus* Went rice were well tolerated with no evidence of significant side effects including neuromuscular functions. Both of them significantly reduced cholesterol after 6 months and 1 year.

**Conclusion:**

*Monascus purpureus* Went rice is safe, effective, and economic treatment strategy for nephrotic dyslipidemia.

## Introduction

Nephrotic hyperlipidemia is a risk factor for the development of systemic artherosclerosis, and may aggravate glomerulosclerosis and enhance the progression of glomerular disease.^1^

Diet intervention should be the first-line of treatment, but it only partially corrects hypercholesterolemia in such patients.^2^ The greatest and the most consistent reductions in low-density lipoprotein (LDL)-cholesterol is seen with 3-hydroxy-3-methylglutaryl-coenzyme A reductase (HMG-CoA reductase) inhibitors. Many studies have shown that *M. purpureus* Went rice contained HMG-CoA reductase inhibitors, large quantities of unsaturated fatty acids, beta-sitosterol, campesterol, and stigmasterol.^3^–^5^ These components are effective in reducing serum lipid.^6^

The lipid-lowering effects of *M. purpureus* - rice food flavor in China and Japan - have been shown in several animal models of hyperlipidemia.^7^ One study showed that *M. purpureus* Went rice significantly reduced LDL-C, total cholesterol, triglycerides, and apolipoprotein B levels, and was well tolerated in patients with hyperlipidemia.^8^,^9^

However, the long-term safety and efficacy of these lipid-lowering strategies are lacking in patients with renal disease.^10^ Treatments of nephrotic dyslipidemia in steroid resistant NS have resulted in a considerable benefit not only for the hyperlipidemia, but also for the nephrotic state.^11^

Prospective controlled studies are needed to evaluate the long-term safety of statins in a large patient population and assess whether reduction in cholesterol decreases the risk for atherosclerosis and inhibits the progression of glomerular disease in patients with NS.^12^ Although statin therapy was considered to be safe in treating nephrotic dyslipidemia on the short-term follow-up studies considering electromyography (EMG),^12^,^13^ long-term studies are lacking for this costly regimen.

We aimed to assess the efficacy and safety of *M. purpureus* Went rice vs. fluvastatin therapy in the management of nephrotic dyslipidemia.

## Materials and Methods

Out of 450 patients with idiopathic nephrotic syndrome screened, 72 were recruited from the nephrology clinic of the Urology and Nephrology Center, Mansoura University for inclusion in this prospective, randomized controlled study for a planned duration of 1 year.

The patients were recruited with the following inclusion criteria: steroid resistant, steroid dependent, and frequently relapsing idiopathic nephrotic syndrome, hypercholesterolemia with no response to an appropriate diet for at least 4 weeks, serum creatinine < 2 mg/dl, recent renal biopsy proved focal segmental glomerulosclerosis (FSGS) or membranoproliferative glomerulonephritis (MPGN). We excluded patients with hepatic disease, muscle disease, history of familial dyslipidemia, diabetes mellitus and those on statins.

This protocol met the requirements of local ethical committee. Upon enrollment, patients were randomly assigned to one of the three treatment groups. The first group comprised 20 cases and was given *M. purpureus*, the second group comprising of 30 cases was given fluvastatin, and the third group (n=22) served as control.

Information regarding the randomized treatment was concealed in sequentially numbered, sealed opaque envelopes. These were opened in the absence of the patients immediately after obtaining informed written consent for participation in the study. The participant physicians were necessarily aware of the randomized treatment in all cases.

Patients were evaluated at start of treatment (control values) and monthly for 6 months (test values). The evaluation included thorough history taking and clinical examination, and following laboratory investigations: Complete urine analysis and 24-h urinary protein estimation, serum creatinine and creatinine clearance using Cockcroft and Gault formula; liver function tests; investigations to exclude secondary causes of glomerulonephritis such as blood sugar, anti-HCV, anti-CMV, anti-HIV, HbsAg, serologic tests for systemic lupus erythematosis rectal mucosal biopsy to exclude schistosomiasis; and total serum cholesterol.

Neurophysiological evaluation was done using the NIHON KOHDEN-evoked response recorder, model MEP-5200, for EMG^14^ and nerve conduction velocity.^15^

### Electromyography

The following muscles were tested:

1. The biceps brachii and the rectus femoris as representative of the proximal muscles of the upper and lower limbs, respectively.
2. The abductor pollicis brevis of the thenar eminence and extensor digitorum brevis as representative of the distal muscles of the upper and lower limbs, respectively.

The electrical potentials were recorded by a bipolar EMG needle electrode (NW-120T). Resting and mild contraction activities were recorded at the interrupted speed and the interference pattern at continuous speed for each muscle.

The mean of at least 20 motor unit action potentials (MUPs), from different sites and depths, gave the duration and amplitude.

### Nerve Conduction velocity

We chose the median nerve and the lateral popliteal nerve for estimating the conduction velocity. The median nerve was stimulated at the antecubital fossa and at the wrist, while the lateral popliteal nerve was simulated at the knee and ankle joints. A bipolar stimulating surface electrode (A-NM-4205) was applied over the nerve and the evoked potentials were recorded by a needle electrode from the abductor pollicis brevis in the upper limb and extensor digitorum brevis in the lower limb. The duration of the stimulus pulse was usually 0.5 ms and the stimulus voltage increased from zero to supramaximal to get the best M wave. A ground electrode was placed between the stimulating and the recording electrodes. Depending on the latency and duration of the evoked potentials, the time bases of the recordings were varied so that the whole pattern of potentials could be displayed on the sweep of the oscilloscope. Usually a time base of 10 ms/cm was needed.

The latency was measured from the start of the stimulus artifact to the onset of the muscle response. The same process was repeated twice, once at the proximal part of the nerve and the other at the distal part. The time difference between the two latencies in milliseconds was obtained.

The conduction velocity was calculated as follows:

Cv = Distance between the two points of stimulation in cm × 10.

Difference between the two latencies in milliseconds = m/sec (meters per second).

The first group received *M. purpureus* Went rice (600 mg twice/day for 1 month then once daily). The second group received oral fluvastatin in a daily dose of 20 mg. The third group received no additional therapy and ranked as a control. After 8 weeks, re-adjustment of the dose was performed according to patient's response (serum cholesterol) and patient's tolerance to the drug. Restriction of dietary cholesterol and proteins was illustrated to every patient by appropriate counseling and repeated reinforcement. Other supportive treatment including angiotensin converting enzyme inhibitor (ACEI) use was comparable in the two groups.

All patients were followed monthly for one year after which they were subjected to thorough clinical and laboratory evaluation.

Statistical analysis was done by an IBM compatible personal computer using the Statistical Package for Social scientists (SPSS) for windows 11.5 (SPSS Inc., Chicago, IL, USA). Qualitative data were displayed in cross tabulation and quantitative data were described in terms of arithmetic mean ± SD. A *P*-value of <0.05 was considered significant.

## Results

The demographic characteristics of all patients were summarized in Table 1. There was no significant difference between the studied groups regarding patients' age, sex, body weight, smoking history, and renal histopathology.

**Table 1:** Demographic characteristics of the studies nephrotic patients

	Fluvastatin (*N* = 30)	Control (*N* = 22)	*Monascus purpureus* (*N* = 20)	*P* value
Mean age (Y)	17.6 ± 7	19.7 ± 7	21.4 ± 14	0.19
Sex (male/female)	13/17	10/12	12/8	0.38
Smokers	3/27	4/18	3/17	0.92
Occupation				
Student	13	9	6	0.51
Worker	5	3	4	0.94
Professional	12	10	10	0.68
Body weight	51.3 ± 26	58.2 ± 25	55.2 ± 24	0.4
Renal pathology				
FSGS	24	14	18	0.58
MPGN	6	8	2	

**Fig. 1:**
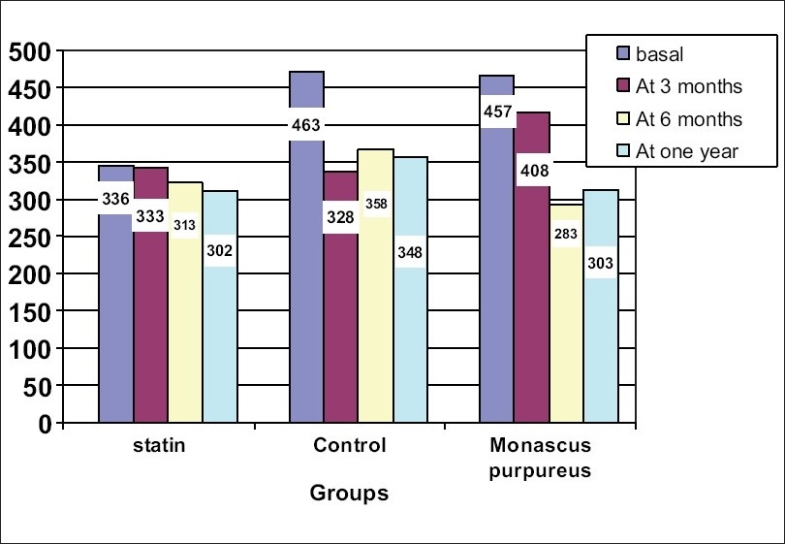
Showed serum cholesterol levels in different groups along follow-up period

Fig. 1 shows the serial value of serum cholesterol in different study groups.

In comparison to baseline values, fluvastatin produced a significant and progressive reduction of serum cholesterol by 35, 38, and 42% at 3 months, 6 months, and after 1 year, respectively (*P* < 0.001). Similar reductions were observed in the *M. purpureus* Went rice group. After 1 year, we observed that serum cholesterol was significantly lower in statin and *M. purpureus* Went rice groups compared to the control group (*P* = 0.003) [Table 2].

**Table 2:** Biochemical characteristics of patients treated by fluvastatin and *Monascus purpureus* Went rice therapies at basal and at the last follow-up of the study

	Statin (*N* = 30)	Control (*N* = 22)	*Monascus purpureus* (*N* = 20)	*P* value, Control *vs* Other groups
Serum CR(mg/dl)				
Basal	1.07 ± 0.4	1.2 ± 0.3	0.85 ± 0.3	0.06
At 6 months	1.03 ± 0.8	0.9 ± 0.6	0.98 ± 0.5	0.53
At 1 year	1.2 ± 0.8	0.99 ± 0.5	0.96 ± 0.3	0.62
S. albumin (gm/dl)				
Basal	1.6 ± 0.7	1.3 ± 0.3	1.67 ± 0.7	0.06
At 6 months	2.1 ± 0.9	1.7 ± 0.8	2.09 ± 1.2	0.56
At 1 year	2.3 ± 0.9	1.7 ± 0.8	2.5 ± 1.6	0.05
Proteinuria (gm/day)				
Basal	8.3	8.8	8.6	0.06
At 6 months	5.2	6.6	5.5	0.055
At 1 year	2.4	7	3.2	0.04
Serum ALT (IU/L)				
Basal	16 ± 9	19 ± 8	15 ± 6	0.5
At 1 year	15 ± 5	17 ± 4.5	17 ± 4	0.9
S. cholesterol (mg/dl)				
Basal	436 ± 102	463 ± 169	457 ± 232	0.31
At 3 months	333 ± 155	328 ± 158	408 ± 239	0.55
At 6 months	313 ± 185	358 ± 178	283 ± 208	0.31
At 1 year	302 ± 171	348 ± 184	303 ± 178	0.003

Serum albumin increased significantly in the statin, *M. purpureus* Went rice groups at 12 months compared to the control group and to the basal values (*P* = 0.05) [Table 2].

In our series, blood chemistry including liver enzymes, bilirubin, alkaline phosphatase, and creatine phosphokinase (CPK) were within normal ranges throughout the study. The mean of CPK showed no significant difference at the start of treatment, after 3 months and at the end of the study.

We observed no clinical evidence of myopathy or neuropathy in our patients who received statins or *M. purpureus* Went rice therapies compared to the basal evaluation.

The amplitude of the MUPs showed no significant changes in the distal muscles after 12 months of fluvastatin or *M. purpureus* Went rice therapies. On the other hand, there was a significant reduction in duration and amplitude of motor action potentials of proximal muscles. However, polyphasicity showed no significant change (*P* > 0.05) [Table 3]. Nerve conduction velocity and terminal latency of the median and lateral popliteal nerves showed no significant changes at 6 months compared to basal values (*P* > 0.05).

**Table 3:** Electromyographic evaluation of *proximal* muscles of the nephrotic patients treated by fluvastatin and *Monascus purpureus* Went rice therapies at basal and at the last follow-up of study

	Fluvastatin (*N* = 30)	*Monascus purpureus* (*N* = 20)	*P* value
			
	Amplitude (mv)	Amplitude (mv)
AMP biceps brachii			
Basal	1487 ± 1510	4250 ± 2500	0.031
Last	1257 ± 1075	3078 ± 1500	0.030
*P* within groups	0.078	0.078	
AMP Q femoris			
Basal	1813 ± 900	3971 ± 2030	0.075
Last	1245 ± 840	4100 ± 2300	0.025
*P* within groups	0.075	0.075	

## Discussion

Hyperlipidemia of the nephrotic syndrome is a risk factor for the development of systemic artherosclerosis, also it may aggravate glomerulosclerosis and enhance the progression of glomerular disease.^1^,^2^,^7^

Pattern of dyslipidemia in our cases is matching with that reported by Wheeler^16^ and Warwick *et al.*^17^ who showed that total plasma cholesterol; TG, VLDL, and LDL were elevated, with variable HDL concentration.

We observed significant reduction of serum cholesterol by 28.8% and 30.2% at 6 months and at 1 year of *M. purpureus* Went rice, possibly due to its HMG-CoA reductase inhibiting properties.^3^–^5^ In contrast, there were no significant changes in serum cholesterol in the control group.

Similar findings were reported by Matzkies *et al.*^18^ who showed a reduction of total cholesterol by 31% and LDL by 29% after 2 months of initiation of fluvastatin treatment (40 mg/day). The relatively small number of cases (10 cases) as well as the large dosage of fluvastatin they used might explain the earlier reduction in LDL and cholesterol they observed. Also, our observation goes hand in hand with the degree of reduction in total and LDL cholesterol reported by Olbricht *et al.*^19^ on using simvastatin with nephrotic patients. Todd and Goa^20^ achieved a 25-30% reduction in plasma LDL within 4 weeks which was maintained with continued treatment by a daily dose of 20-40 mg fluvastatin. Jokubaitis^21^ reported similar findings by fluvastatin in a dose of 40 mg/day.

In this study, we succeeded in reducing cholesterol by the same degree after 6 months of fluvastatin (20 mg per day) and - for the first time in nephrotic patients - by *M. purpureus* (600 mg per day) therapies, without side effects and with relatively lower dosage.

In the same direction, Lin *et al.*^8^ reported short-term efficacy and safety of *M. purpureus* went rice in treating hyperlipidemia.

Interestingly, a significant reduction in proteinuria was observed in the statin and *M.* Went rice treated patients, but not in the control group. Matzkies *et al.*^18^ in a similar study - with statin failed to demonstrate such favorable effect. This may be explained by the fact that Matzkies' patients were of heterogeneous pathologic types while most of our patients were suffering from FSGS.

In the same direction, Hattori *et al.*^22^ reported that there was an improvement in renal function and proteinuria in drug resistant NS secondary to FSGS, by LDL apheresis combined with pravastatin. In placebo-controlled study performed in Hong-Kong over 2 years, it was suggested that despite no significant effect on proteinuria a decline in renal function was attenuated by lovastatin, particularly over the second year of the study.^23^ Chan *et al.*^24^ reported that in lovastatin-treated nephrotics with relatively good pretreatment renal function, glomerular filtration rate (GFR) increased at the end of 6 months treatment.

In contrast, Matzkies *et al.*^18^ reported a significant rise in serum creatinine in their patients despite using fluvastatin in a dose of 40 mg/day. Again, the heterogeneity of pathologic types of their patients explains the difference in their results.

In this study, the significant reduction of creatinine clearance among control group 6 months onward compared to the nonsignificant change of creatinine clearance in *M. purpureus* Went rice and statin-treated groups, both suggested the protective effect of lipid-lowering agents on kidney function. Also, the electromyographic data showed a significant decrease in the amplitude and duration of MUP in the proximal muscles only with statin treatment compared to basal values. These changes were not observed in *M. purpureus*. However, this reduction was not excessive and there was no other electromyographic evidence of myopathy. In addition, there were no clinical findings of myopathy or elevated serum CPK.

Peters^25^ similarly noted that drug-related myopathy and rhabdomyolysis have not been reported with fluvastatin on the basis only of clinical findings and elevated skeletal muscle enzymes. In the same direction, Jokubaitis^21^ found no notable increase in serum CPK or liver enzymes, and no cases of clinically evident myopathy. On the other hand, Careless and Cohen^26^ reported that statins and fibrates were associated with a variety of rheumatic problems including proximal myopathy, diagnosed on a clinical basis and confirmed by high serum CPK. Jacquet *et al.*^27^ also reported that the frequency of severe side effects such as myopathy amounted to 1 per 1000 prescriptions with cholesterol-lowering drugs in current use. De Pinieux *et al.* ascribed the myopathic side effects of statins to mitochondrial dysfunction as the blood lactate/pyruvate ratio is high.^28^ These reports regarding the safety of statins were based only on clinical data and muscle enzyme evaluation.

Jacobson *et al.*^29^ reported increase in liver enzymes while Olbricht *et al.*^19^ and Matzkies *et al.*^18^ reported satisfactory tolerance of statin in their nephrotics.

In agreement with what was reported by Lin *et al.*^8^, *M. purpureus* Went rice was well tolerated in patients with hyperlipidemia. Moreover, we found that *M. purpureus* Went rice was not only an efficacious modality of treatment that targeted the nephrotic dyslipidemia with the same potency like statin, but also achieved 50% cost reduction in comparison to fluvastatin. From this study, we can conclude that *M. purpureus* Went rice is safe, effective, and economic treatment strategy for nephrotic dyslipidemia.
